# Optimization of methane sensing response of a ZnO-graphene composite using the response surface method: sensing area and annealing temperature

**DOI:** 10.1039/d5ra07597a

**Published:** 2025-12-15

**Authors:** M. Adzhim Sayuti, Siti Amaniah Mohd Chachuli, Omer Coban, N. H. Shamsudin

**Affiliations:** a Fakulti Teknologi dan Kejuruteraan Elektronik dan Komputer, Universiti Teknikal Malaysia Melaka, (UTeM) Jalan Hang Tuah Jaya 76100 Durian Tunggal Melaka Malaysia sitiamaniah@utem.edu.my; b Faculty of Engineering, Ataturk University Erzurum Turkiye; c Fakulti Teknologi dan Kejuruteraan Elektrik, Universiti Teknikal Malaysia Melaka, (UTeM) Jalan Hang Tuah Jaya 76100 Durian Tunggal Melaka Malaysia

## Abstract

Methane traps heat 25 times more than carbon dioxide and is highlighted as the second most potent greenhouse gas, contributing to climate change. As the methane level grows, the impact on the Earth's climate becomes more severe, and exposure to high levels can lead to adverse consequences for the human health, causing symptoms like changes in breathing and heart rate, numbness, and death in case of prolonged and high exposure. To address these concerns, this study focuses on the optimization of a ZnO-graphene composite in gas sensors for methane sensing at room temperature using the response surface method (RSM). RSM was conducted using the Design Expert 13 software by optimizing two parameters: sensing area and annealing temperature. Ten samples of ZnO-graphene gas sensors were fabricated based on the sensing layer area (1 cm^2^–4 cm^2^) and annealing temperature (100–200 °C). The ZnO-graphene gas sensor was fabricated using a screen-printing technique on a Kapton film by applying silver paste (Ag) as the interdigitated electrode and ZnO-graphene as the sensing layer. The optimization using the RSM highlighted that the experimental model was significant with an *R*^2^ value of 0.8871. Results revealed that the sensing layer area has more influence on the gas sensor sensitivity than the annealing temperature. The optimized model showed that an area of 4 cm^2^ and an annealing temperature of 100 °C are the optimal parameters, with a sensitivity value of approximately 0.968167 × 10^−3^ for 5000–7000 ppm of methane.

## Abstract 2

Three-dimensional plots of sensitivity based on the interactive effects of sensing area and annealing temperature. Optimization using RSM highlighted that the experimental model was significant with an *R*^2^ value of 0.8871.

## Graphical abstract

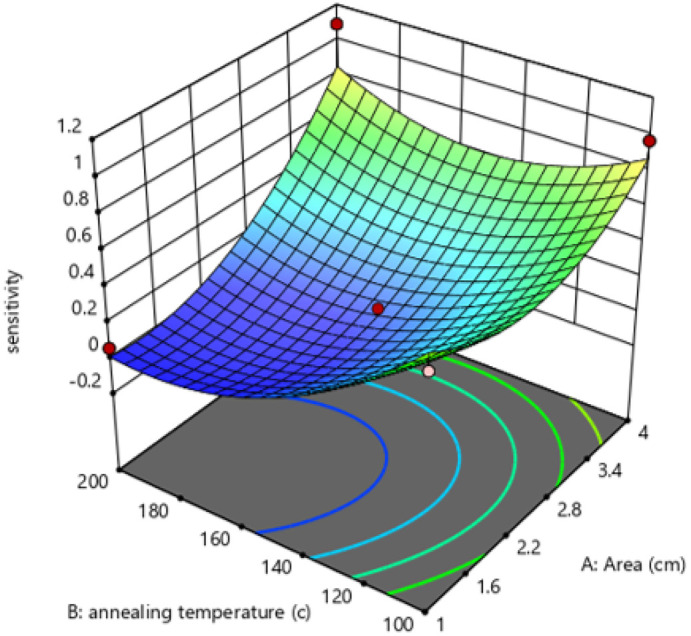

## 1 Introduction

Methane (CH_4_) is a colorless and flammable gas commonly found in natural gas.^1^ As the second most potent greenhouse gas, methane contributes about 20% to climate change, trapping atmospheric heat nearly 25 times more effectively than carbon dioxide.^2^ Although it accounts for only a small percentage of atmospheric gases, approximately 0.0002% by volume (2 ppmv),^3^ its impact on climate change is significant since the concentration keeps increasing at a rate of about 1% per year.^4^ As methane concentration increases, its effects on the Earth's climate become more pronounced, and exposure to high methane levels can lead to adverse consequences for the human health.^4^ According to the Public Health England, exposure to 2000 ppm methane levels might reduce the amount of oxygen in the air, leading to various symptoms, including changes in the breathing and heart rates, loss of body balance, numbness, unconsciousness, vision disturbances, nausea, vomiting, and headaches. If exposure is large or continued for a longer period, it might lead to death.^5^ Furthermore, it can lead to explosion hazards when methane concentrations reach 50 000 ppm (5%) and higher.^6^

The development of sophisticated gas sensors capable of accurately and instantly detecting methane (CH_4_) emissions is of significant importance in order to address these concerns. Gas sensors are employed in many other sectors and applications, where they are used to detect dangerous gases that contribute to quality, safety, and environmental issues simultaneously. The literature indicates that metal oxide semiconductors (MOS) have attracted much interest in the study of methane gas detection. Common examples that are well-known for their excellent gas sensing capabilities are zinc oxide (ZnO),^2,7^ tin oxide (SnO_2_),^8,9^ vanadium pentoxide (V_2_O_5_),^1^ and indium oxide (In_2_O_3_).^10^ Zinc oxide (ZnO) was chosen as the sensing material for the gas sensor in this study because of its exceptional electrical, chemical, and physical properties, which make it a very effective methane detector.^11^ ZnO is an n-type semiconductor with a wide band gap of approximately 3.4 eV, a high exciton binding energy of around 60 meV, and a high electron mobility of 200 cm^2^ V^−1^ s^−1^. It has excellent chemical and thermal stability, along with a strong photoelectric response, making it a promising material for gas sensors.^12^ Studies^2^ demonstrated that ZnO spheres exhibit excellent methane-sensing performance, achieving a 20.577% response towards 5000 ppm methane with a rapid response time of 6 seconds under UV light at room temperature. SnO_2_ and V_2_O_5_ were not included in this study due to their inherent limitations. Issues such as a lack of selectivity among various reducing gases and an initial conditioning phase, during which its resistance gradually wanders, limit the reliability of SnO_2_ for several gas-detection applications.^13^ But unlike other vanadium compounds, V_2_O_5_ is highly toxic and can cause major health risks, including eye irritation, severe respiratory issues, and a metallic taste.^14^ These characteristics make both materials less suitable for the scope of this study.

Research in gas sensor technology has made significant advancements, with research focused on improving sensor performance through material innovation, sensor design, and optimization techniques.^15–17^ Several studies have focused on improving gas sensing capability by modifying catalyst nanoparticles or nano-structuring sensitive metal oxide materials.^18^ For example, La doping in ZnO enhanced gas sensing capabilities by decreasing crystallite size, improving surface roughness, and reducing the optical band gap from 3.275 eV to 3.125 eV. The 4.0 at% La-doped ZnO sensor demonstrated a 114.22% response to CO_2_ at 200 sccm, with a response time of 24.4 s and recovery time of 44 s. These improvements, which result from its increased surface area and active patches, demonstrate its potential for efficient gas detection.^19^ Graphene is an isolated single layer of carbon hexagons with sp^2^-hybridized C–C bonds and π-electron clouds, which makes this material significant in engineering due to its unique structural and physical properties and potential technological applications.^20^ Graphene is often used either in its pristine form or combined with metals, metal oxides, conducting polymers, and other systems due to its surface functional groups, which enhance sensitivity, selectivity, room temperature operations, surface area, conductivity, and response speed.^21^ Past studies have shown that gas sensors based on graphene can operate at room temperature.^22^ Thus, graphene nanoflakes were used to dope ZnO to make a ZnO-graphene composite and enhance the sensing mechanism of the gas sensor in this study.

Optimization is a technique used to enhance sensor performance factors, such as sensitivity, noise, and power consumption.^23^ Several optimization techniques are widely used, which are multivariate,^24^ factorial,^25^ Taguchi Method,^26^ and Response Surface Methods (RSM).^17^ Among optimization techniques, RSM is a popular experimental design for optimization purposes due to its ability to leverage a relatively small number of trials cost-effectively.^27^ For example, RSM was employed in optimizing charge collection in solar cells^28^ and gas sensor sensitivity.^17^ By determining interaction effects of independent input parameters and utilizing data-driven model equations, RSM effectively illustrates the various combinations of factors influencing a process or product outcome. Moreover, RSM allows for the approximation of both 5 experimental and numerical responses (−*α*, −1, 0, +1, +*α*), maintaining a high level of efficiency in terms of cost and time. In comparison to other methods, like Taguchi and one-factorial, RSM emerges as more promising in mathematical modeling for forecasting responses.^27^ Thus, the present study is focused on optimizing ZnO-graphene gas sensors for methane sensing using RSM.

The overall goals of this study are to optimize the ZnO-graphene gas sensor to achieve high sensitivity to methane by fabricating gas sensors using various sizes of the sensing layer and annealing at different temperatures based on an experimental design matrix. RSM, as an optimization method, was chosen, and 10 samples of ZnO-graphene gas sensors were fabricated based on the proposed specifications. All the gas sensors were deposited onto Kapton films using the screen-printing method. The results showed that RSM can be used as an experimental tool for optimization in gas sensing applications. It was also concluded that the annealing temperature had more impact on the sensing response than the area of the sensing layer.

## 2 Experimental works

### 2.1 Materials

Ethyl cellulose (48.0–49.5% (w/w), ethoxyl basis), alpha-terpineol (97%), ZnO powder, and graphene nanoflakes (>99%) were purchased from Sigma-Aldrich, Acros Organics, Chemiz (M) Sdn Bhd, and BT Corp, respectively. All materials were used without purification.

### 2.2 Material characterizations

Field emission scanning electron microscopy (FESEM) and X-ray diffraction (XRD) characterizations were carried out on a ZnO thick film on the Kapton film that was annealed at different temperatures: 79 °C, 100 °C, 150 °C, 200 °C, and 220 °C. FESEM (model: Hitachi SU 5000) was performed at 10 kV and 30k magnification to observe the ZnO structure. The X-ray Diffraction (XRD) (model: X'pert High Pro Panalytical) studies were then carried out for thick film in 30 min over a 2*θ* range between 3° and 90°.

### 2.3 Experimental design

RSM analyses the relationship between two parameters (area of the sensing layer and annealing temperature) in order to maximize the performance of ZnO-graphene gas sensors. This study utilized Design Expert 13.0.5.0 (STATEASE, Inc., Minneapolis, USA) software to perform experiments and fit a polynomial model for sensor sensitivity to methane gas, defined as the slope of the sensing response *versus* methane concentration plot. Table 1 summarizes the experiment design parameters. The range for parameter *A* (area) was set between 1 cm^2^ and 4 cm^2^, with axial points extending from 0.37868 cm^2^ to 4.62132 cm^2^ and parameter *B* (annealing temperature) was set between 100 °C and 200 °C, with axial points extending from 79.2893 °C to 220.711 °C. Central Composite Design (CCD) or Box–Wilson method is a common method for RSM.^29^ In this study, a Central Composite Design (CCD) was used, requiring 10 experiments: 2 at the central point, 4 at axial points, and 4 at factorial points, covering five levels (−*α*, −1, 0, +1, +*α*). The values of each experimental design variable were suggested by the software and are shown in Table 2. The ten samples were fabricated based on the suggested values proposed in Table 2.

**Table 1:** Experimental range and the levels of independent variables

Name	Units	Low	High	−Alpha	+Alpha
*A*: area	cm^2^	1	4	0.37868	4.62132
*B*: annealing temperature	°C	100	200	79.2893	220.711

**Table 2:** Experimental design variables for the optimization of ZnO-G

Std	Run	Space type	Factor 1	Factor 2
			*A*: area (cm^2^)	*B*: annealing temperature (°C)
6	1	Axial	4.62132	150
3	2	Factorial	1	200
9	3	Center	2.5	150
10	4	Center	2.5	150
8	5	Axial	2.5	220.711
5	6	Axial	0.37868	150
4	7	Factorial	4	200
1	8	Factorial	1	100
2	9	Factorial	4	100
7	10	Axial	2.5	79.2893

The model for the sensitivity response is shown in eqn (1). In this equation, sensitivity is the response variable, *x*_*i*_ and *x*_*j*_ are the factors of choice for optimization, and *β*_0_ is the constant coefficient. The coefficients of each factor are shown as linear, quadratic, and interacting with *β*_*i*_, *β*_*ii*_, and *β*_*ij*_, respectively. The last coefficient in equation (*e*_*i*_) is related to the predicted error in the model. The study determined optimal conditions for *A* (area of the sensing layer) and *B* (annealing temperature), which improve sensor sensitivity and demonstrate the effectiveness of RSM for gas sensor optimization.

1 Sensitivity = *β*_0_ +∑β_*i*_*x*_*i*_ + ∑*β*_*ij*_*x*_*i*_*x*_*j*_ + ∑*β*_*ii*_*x*_*i*_^2^ + *e*_*i*_

### 2.4 Preparation of ZnO-graphene paste

**Fig. 1:**
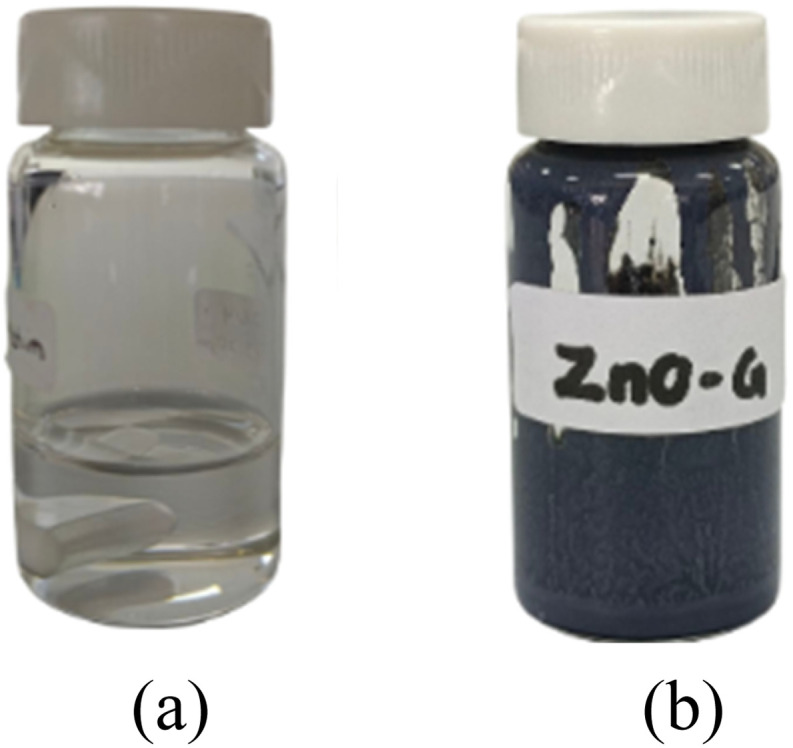
Sensing layer preparation. (a) Binder and (b) ZnO-G paste.

The sensing layer for ZnO-graphene is prepared in two major steps: preparation of the binder and preparation of ZnO doped with graphene paste. Ethyl cellulose and terpineol were employed as the organic binder in this work. To create a homogeneous binder for the sensing material, 95 wt% of terpineol and 5 wt% of ethyl cellulose were mixed using a magnetic stirrer. The mixing was performed for 36 hours at 300 rpm (rpm) and 40 °C temperature. Furthermore, the sensing layer of the sensor was prepared by mixing 80 wt% ZnO powder and 20 wt% graphene powder. The mixture was combined with 40 mL of acetone and sonicated for 30 minutes at 50 °C. The sonication process was carried out in an ultrasonic cleaner (frequency: 40 kHz, brand: SMITH3D, model: CR-010S, ultrasonic power: 60 W, heating power: 150 W). After sonication, the mixture was annealed at 100 °C until it dried. The dried mixture was then ground to form a fine powder. The sensing layer was created by combining the ZnO-graphene (ZnO-G) powder and binder in a 40 : 60 ratio and stirring at 40 °C and 80 rpm. It took 24 hours to obtain a homogeneous and viscous paste from the mixture. Fig. 1 shows the produced binder and ZnO-G paste.

### 2.5 Preparation of the ZnO-graphene gas sensor using screen printing technology

**Fig. 2:**
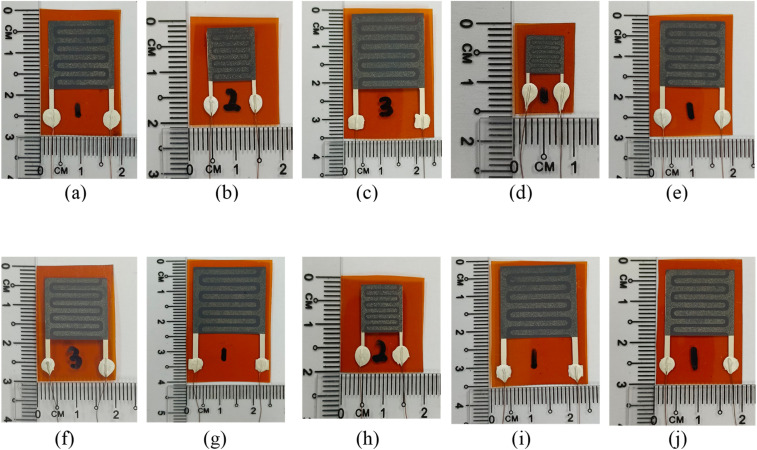
Fabricated ZnO-G gas sensor using screen-printing technique (a) ZnO-G_S1, (b) ZnO-G_S2, (c) ZnO-G_S3, (d) ZnO-G_S4, (e) ZnO-G_S5, (f) ZnO-G_S6, (g) ZnO-G_S7, (h) ZnO-G_S8, (i) ZnO-G_S9 and (j) ZnO-G_S10.

Kapton is the chosen substrate for this gas sensor fabrication because of its flexibility and ability to work at room temperature. The area of the sensing layer, as a parameter for optimization using Response Surface Method (RSM), was set in the range from 1 cm^2^ to 4 cm^2^ in Design Expert 13 software, influencing the size of the substrate. The Kapton film was cut based on the size suggested in Table 2. To ensure the Kapton films were clean, isopropyl alcohol was wiped onto the surface of the Kapton film. The ZnO-G gas sensor consists of two layers: an interdigitated electrode and a sensing layer. The stencil for the interdigitated electrode and sensing layer was made from polyester, and the thickness is ±10 µm with a mesh count of P120-34Y. Firstly, an interdigitated electrode was printed onto the substrate using a screen-printing technique using silver paste (Ag), followed by 30 minutes of annealing at 150 °C using an oven under ambient air. ZnO-G paste is layered on top of the interdigitated electrode as the sensing material of the gas sensor with a certain size and annealed at a specific temperature, as given in Table 2, for 1 hour in the oven under ambient air. After the deposition of the interdigitated electrode and sensing material on the Kapton film was completed, fine copper wires cleaned using sandpaper, about 3 cm in length, are utilized in both these processes to establish an electrical connection. The fabrication process of the gas sensor was adapted from ref. in 30. Ten samples of ZnO-G gas sensors were fabricated, as per optimization using RSM requirements and are displayed in Fig. 2.

### 2.6 Current measurement of the ZnO-G gas sensor

**Fig. 3:**
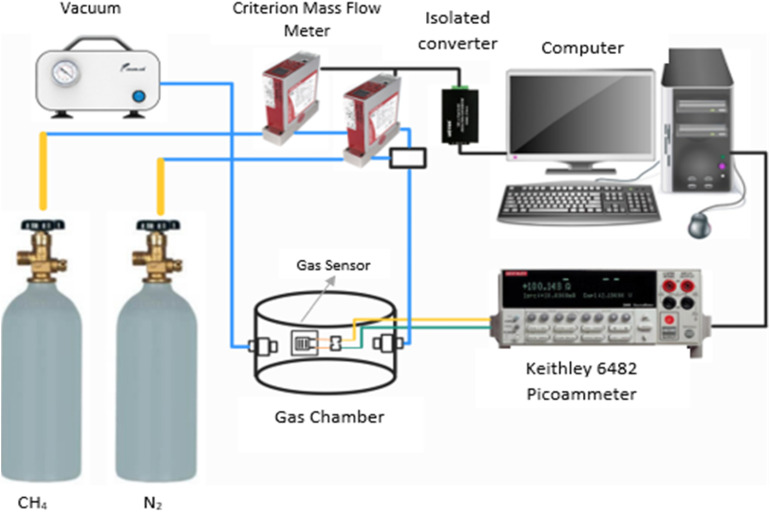
Experimental setup of the proposed gas sensor for methane.

The experimental setup for the measurement of the ZnO-G gas sensor for methane is illustrated in Fig. 3. The computer, Horiba mass flow controller, and source meter (Keithley 6482) are all linked to the gas chamber through their respective cable connections. The Horiba mass flow controller controls the flow rate of the gas through the sensor. The LabVIEW software was used to monitor the changes in the current (output). The gas sensor was placed inside the gas chamber during the current measurement process. Gas sensing measurements were performed in a gas chamber by exposing the ZnO-G gas sensor to methane concentrations of 5000 ppm and 7000 ppm. Initially, nitrogen acts as a carrier gas and flows to the gas sensor for 20 minutes for stabilization. Then, the gas sensor is exposed to methane gas in the gas chamber for 20 minutes to determine its response. Subsequently, nitrogen gas is allowed to flow again for 20 minutes, during which the recovery time is analyzed.

## 3 Results and discussion

### 3.1 Characterization of the sensing layer using field emission scanning electron microscopy (FESEM) and X-ray diffraction (XRD)

**Fig. 4:**
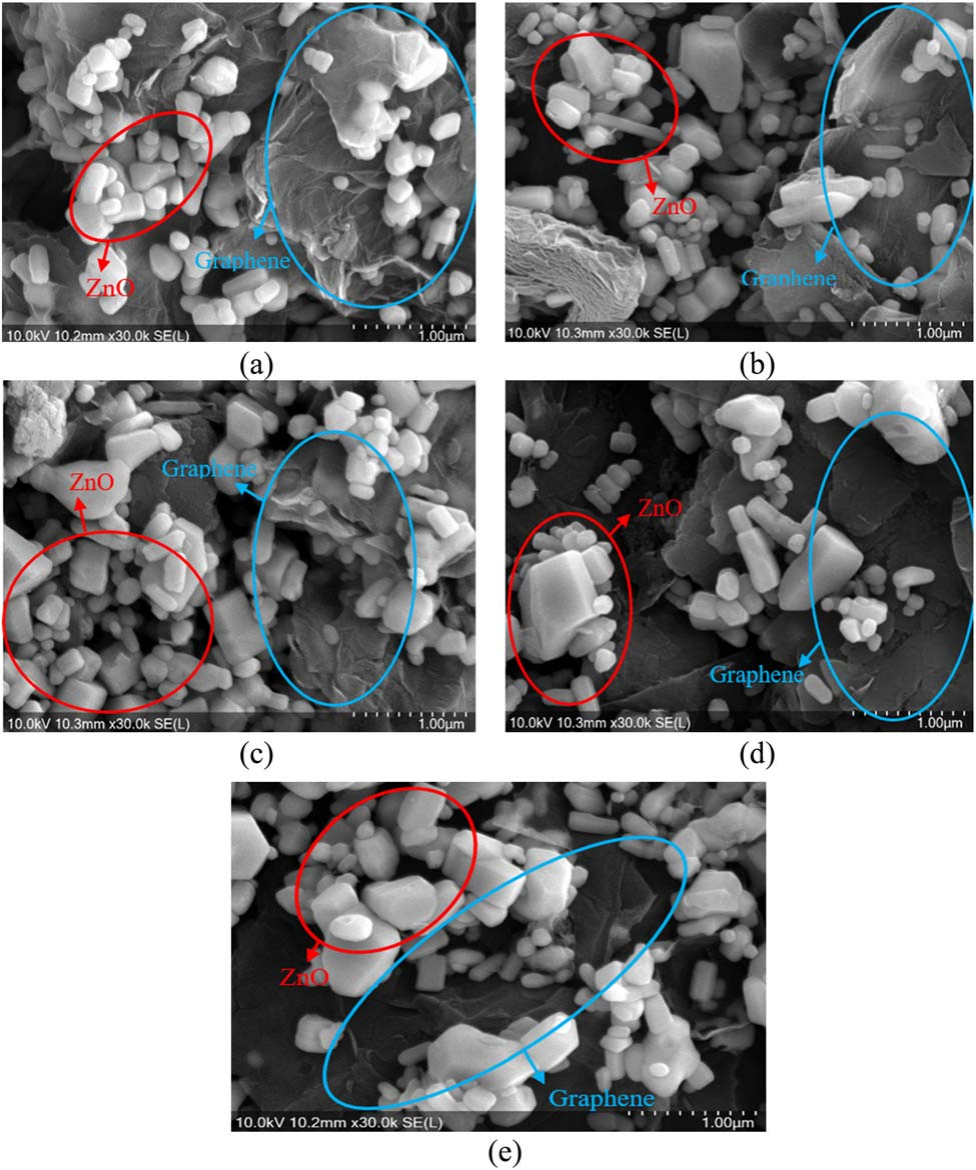
Surface morphology of ZnO-G using FESEM analysis at specific annealing temperatures of (a) 79 °C, (b) 100 °C, (c) 150 °C, (d) 200 °C, and (e) 220 °C.

**Fig. 5:**
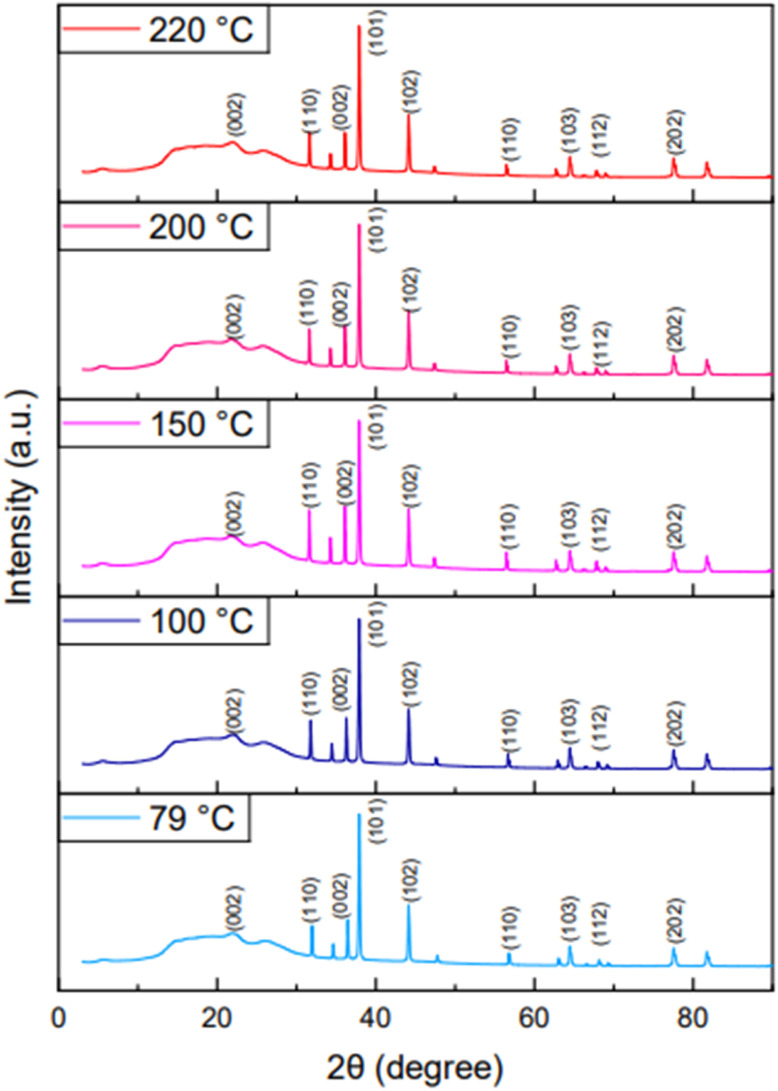
XRD spectra of ZnO-G at specific annealing temperatures.

FESEM and XRD characterizations of the surface of the gas sensor on the Kapton film after annealing at a specific temperature (Table 2) are presented in Fig. 4 and 5. It can be seen that ZnO-G exhibited a hybrid morphology in which graphene is present as nanoflakes, as reported previously,^22^ while ZnO produced a variation of nanostructured morphologies like nanorods or nanosheets. The surface morphology of graphene demonstrates a larger morphology compared to ZnO, and it maintains a stable structure with the large surface area even at high temperatures. ZnO shows morphological changes as the annealing temperature increases, showing that the lowest temperature produced less-defined nanostructures, while higher temperatures demonstrated more crystalline forms, such as nanorods or nanosheets.^31^ The surface morphology of the ZnO-G material is shown in Fig. 4. Graphene nanoflakes were detected in the sensing material, thus verifying that the doping process was successful in ZnO-G. The red circle indicates ZnO particles, while the blue circle indicates graphene nanoflakes.

Fig. 5 shows the XRD spectra of the sensing layer after annealing treatment. Prior research revealed diffraction patterns with significant peaks at 2*θ* values between 30° and 80°, which correlate to the ZnO hexagonal wurtzite structure.^32,33^ In this study, the material was verified to be ZnO since the peak was found in the same range. According to,^34^ the peaks observed at 2*θ* = 26° belonged to graphene. However, the peak detected in Fig. 5 at 2*θ* = 22° shows the presence of graphene in the sensing material, which is slightly shifted. This phenomenon may be caused by factors such as instrument settings and sample preparation.^35^ The XRD analysis did not show a significant impact due to the different annealing temperatures. A relatively similar peak occurred at 2*θ* = 22°, corresponding to a small intensity (002) peak.

### 3.2 Current–voltage characteristics of the ZnO-G gas sensor

**Fig. 6:**
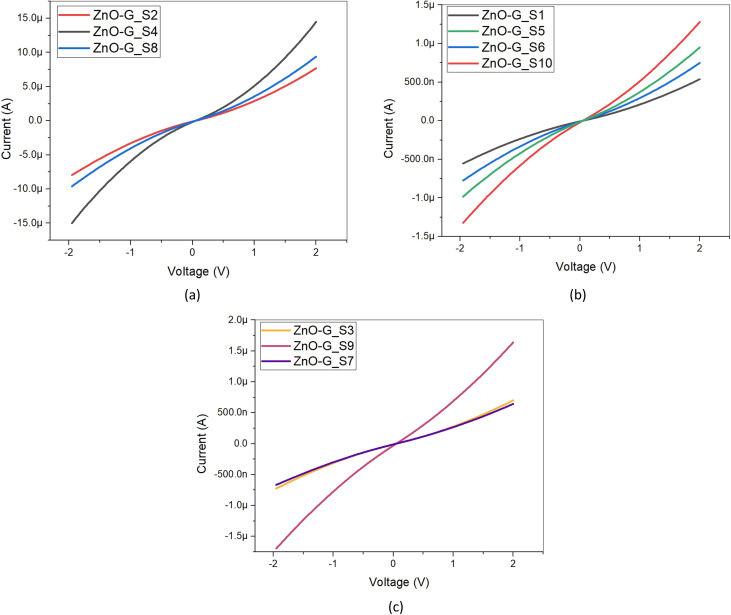
Current–voltage curves of the ZnO-G gas sensor, ranging from −2 V to 2 V. (a) 0.38 cm^2^ and 1 cm^2^, (b) 2.5 cm^2^ and (c) 4 cm^2^ and 4.7 cm^2^.

Current voltage (*I*–*V*) characteristics of the ZnO-G gas sensor were measured using a two-point probe method with a source meter (Keithley 6482). LabVIEW software was used to set the supply voltage and monitor the output current of the gas sensor. Fig. 6 shows the *I*–*V* characteristic of the fabricated ZnO-G gas sensor at a voltage range from −2 V to 2 V, which has been separated into three categories: small size (sample with area 0.38 cm^2^ and 1 cm^2^), medium size (sample with area 2.5 cm^2^), and large size (sample 4 cm^2^ and 4.7 cm^2^). Table 3 shows the resistance and conductivity values for the ten experimental runs of the ZnO-G gas sensor. All the ZnO-G gas sensors produced linear characteristics, following Ohm's law,^36^ but with a slight curvature observed at a lower area and annealing temperature. A more linear characteristic indicates good ohmic contacts between the sensing layer and electrodes.^37^ However, this curve pattern behavior does not hinder but permits their exposure to methane.

**Table 3:** Resistance and conductivity values of the ZnO-G gas sensor at 2 V

Area (cm^2^)	Anneal. temperature (°C)	Sample name	Resistance (MΩ)	Conductivity (×10^−6^ Ω m)
0.38	150	ZnO-G_S4	0.154	6.4935
1	100	ZnO-G_S2	0.283	3.5336
1	200	ZnO-G_S8	0.231	4.3290
2.5	79	ZnO-G_S1	3.993	0.2504
2.5	150	ZnO-G_S5	2.249	0.4446
2.5	150	ZnO-G_S6	2.853	0.3505
2.5	220	ZnO-G_S10	1.654	0.6046
4	100	ZnO-G_S3	3.036	0.3294
4	200	ZnO-G_S9	1.267	0.7893
4.7	150	ZnO-G_S7	3.240	0.3086

From Table 3, it can be seen that the highest resistance of ZnO-G gas sensors was produced by ZnO-G_S1 (3.993 MΩ), which is because of the lowest annealing temperature on the sensing layer, and the lowest resistance was produced by ZnO-G_S4 (0.154 MΩ), which is because of the smallest sensing area. Conductivity is inversely related to resistance; thus, low resistance will produce high conductivity. Therefore, ZnO-G_S4 generated the highest conductivity, while ZnO-G_S1 generated the lowest conductivity. The conductivity of a gas sensor indicates the adsorption of ionized oxygen during carrier gas flow, wherein higher conductivity will attract more ionized oxygen attached to the surface of the gas sensor. Thus, it will increase the interaction of oxygen and methane molecules, therefore enhancing the methane sensing response.

### 3.3 Sensing characteristics of the ZnO-G gas sensor to methane

**Fig. 7:**
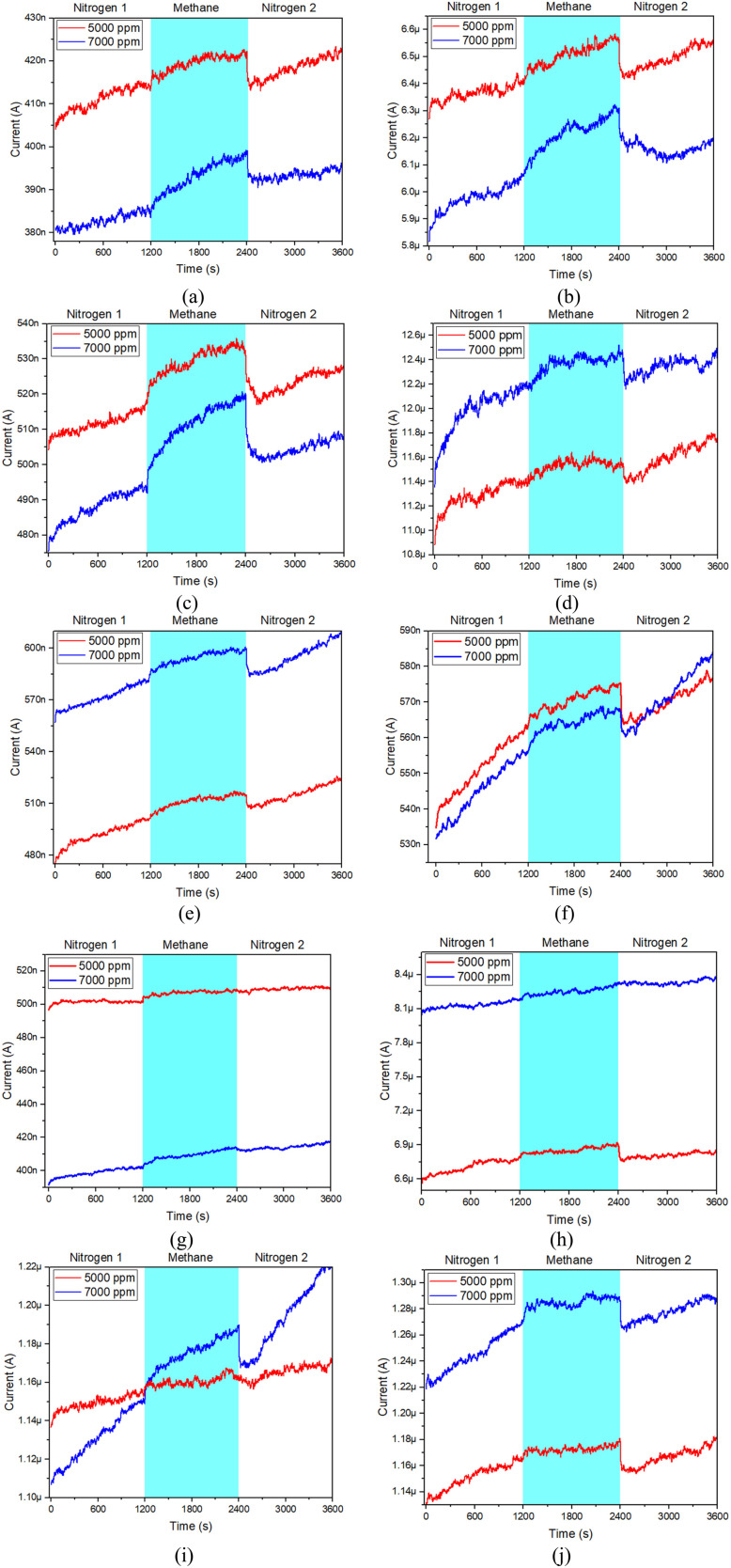
Current measurement of the ZnO-G gas sensor to methane: (a) ZnO-G_S1, (b) ZnO-G_S2, (c) ZnO-G_S3, (d) ZnO-G_S4, (e) ZnO-G_S5, (f) ZnO-G_S6, (g) ZnO-G_S7, (h) ZnO-G_S8, (i) ZnO-G_S9, and (j) ZnO-G_S10.

**Fig. 8:**
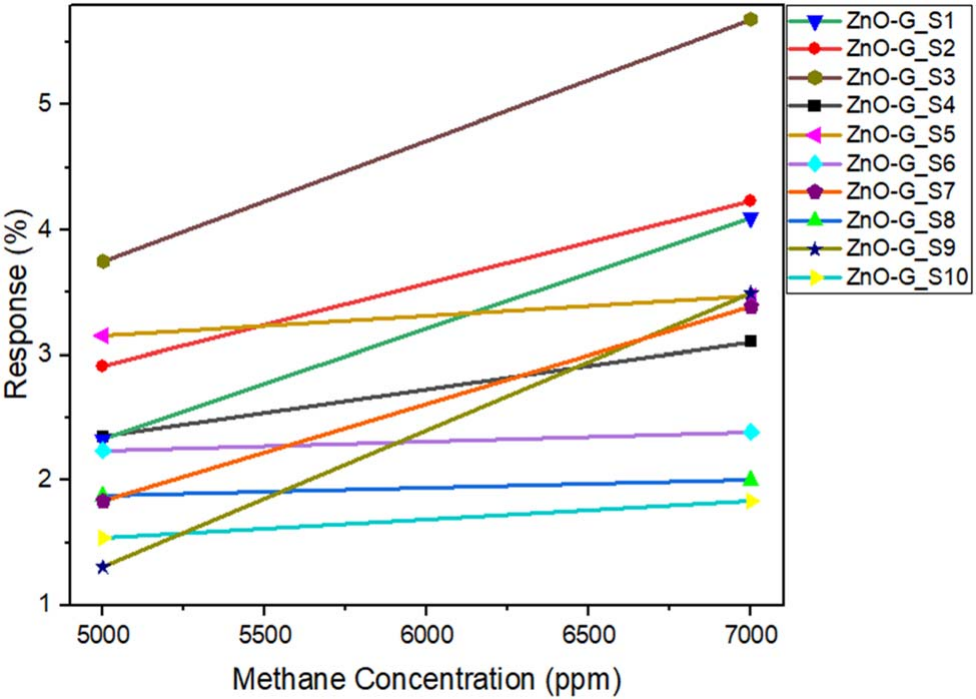
Sensitivity graph of ZnO-G gas sensors.

Fig. 7 and 8 show the measurement of current and sensitivity of ZnO-G gas sensors to methane concentrations of 5000 ppm and 7000 ppm. The characteristics of the gas sensor were evaluated in terms of sensing response and sensitivity properties. The response of the ZnO-G gas sensor was evaluated using the formula as follows:^38^2
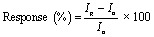
where *I*_g_ is the current during methane flow, and the value is taken at the maximum value of current during methane exposure, while *I*_a_ is the current during nitrogen flow, and the value is taken at the stable or minimum value during nitrogen exposure. The sensitivity value was calculated by finding the slope of the response graph for 5000 ppm and 7000 ppm of methane.^12^

The results indicated that all ZnO-G gas sensors responded to methane as n-type gas sensors, with increased current when exposed to the target gas (methane) and the current also increased when exposed to the carrier gas (nitrogen), as shown in Fig. 7. This result also verified that the ZnO-G used in this study is an n-type semiconductor based on the sensor response. These results were similar to those reported in another study.^39^ The gas sensor was exposed twice: the first exposure was for 5000 ppm and the second exposure was for 7000 ppm of methane. It can be observed that the ten samples of ZnO-G gas sensors showed a similar pattern when exposed to methane, and the response increased with increasing methane concentration.

The sensitivity values for all the ZnO-G gas sensors ranged from 0.062598 × 10^−3^ to 1.090000 × 10^−3^. The graph in Fig. 8 shows that the sensors produced the highest sensitivity when exposed to 7000 ppm of methane concentration. The increase, depending on the response and methane concentration, demonstrates the linearity of sensitivity. The more linear the slope, the more sensitive the ZnO-G gas sensor to the target gas.^40^ The graph highlights that the highest slopes were generated by ZnO-G_S3 and ZnO-G_S9 gas sensors, showing that the fabricated ZnO-G gas sensor is a good candidate for higher sensitivity.

### 3.4 Optimization using response surface method

#### 3.4.1 Second order model and statistical analysis

In the central composite design (CCD) experiment, the sensitivity data for each ZnO-G is listed in Table 4. The equation that sets the correlation between gas sensor sensitivity and process variables was found to be a quadratic polynomial. Eqn (3) displays the final empirical model for the sensor sensitivity yield based on the coded values after eliminating insignificant terms. The model and equation obtained in this study are similar to those from studies in ref. 17 and 41 which focused on optimizing the sensitivity and performance of gas sensors.

3 Sensitivity = 4.07132 − 0.797813 *A* − 0.038303 *B* + 0.002388 *AB* + 0.119610 *A*^2^ + 0.000095 *B*^2^

**Table 4:** Design experiment matrix based on central composite design (CCD)

Std	Run	Space type	Factor 1	Factor 2	Response
			*A*: area (cm^2^)	*B*: annealing temperature (°C)	sensitivity (×10^−3^)
6	1	Axial	4.62132	150	0.777319
3	2	Factorial	1	200	0.062598
9	3	Center	2.5	150	0.073488
10	4	Center	2.5	150	0.154396
8	5	Axial	2.5	220.711	0.144838
5	6	Axial	0.37868	150	0.379759
4	7	Factorial	4	200	1.090000
1	8	Factorial	1	100	0.657077
2	9	Factorial	4	100	0.968167
7	10	Axial	2.5	79.2893	0.886734

The correlation coefficient value was used to assess the model's quality. From eqn (2), the *R*^2^ and adjusted *R*^2^ values are 0.8871 and 0.7459, respectively. This indicates that the relationship between the independent variables (area of the sensing layer and annealing temperature) and the response (sensitivity) is reasonably well-explained by the regression model. The standard deviation for the model was 0.2022. Based on the study in ref. 17, the model will perform better if the *R*^2^ value is closer to unity and the standard deviation is smaller, as it will produce a predicted value for the response that is more in line with the actual value. The predicted *R*^2^ of 0.2038 is significantly lower than the adjusted *R*^2^ of 0.7459, indicating that the model is not predictive accurate for new data (unseen data); however, the model does a good job of fitting the training data. Adeq precision measures the signal-to-noise ratio. In this study, the ratio of 6.2205 is demonstrated as an adequate signal, which is desirable since the ratio is greater than 4. The *R*^2^, adjusted *R*^2^, predicted *R*^2^, and standard deviation of the model are shown in the fit statistics in Table 5.

**Table 5:** Fit statistics of the model

Std. Dev.	0.2022	*R* ^2^	0.8871
Mean	0.5194	Adjusted *R*^2^	0.7459
C.V.%	38.93	Predicted *R*^2^	0.2038
		Adeq Precision	6.2205

The adequacy of the model was further justified through analysis of variance (ANOVA). The optimization suggested a quadratic model for this experiment. The ANOVA for the quadratic model for ZnO-G gas sensor sensitivity to methane is listed in Table 6. These results suggest that the proposed model is significant, which supports the optimization results. Although the lack of fit was revealed as not significant, this result may be due to fewer samples used for training this model.

**Table 6:** ANOVA for the quadratic model

Source	Sum of squares	df	Mean square	*F*-Value	*p*-Value	
Model	1.28	5	0.2569	6.28	0.0496	Significant
*A*-Area	0.4516	1	0.4516	11.05	0.0293	
*B*-Annealing temperature	0.2895	1	0.2895	7.08	0.0563	
*AB*	0.1283	1	0.1283	3.14	0.1512	
*A* ^2^	0.3311	1	0.3311	8.10	0.0466	
*B* ^2^	0.2584	1	0.2584	6.32	0.0658	
Residual	0.1635	4	0.0409			
Lack of fit	0.1603	3	0.0534	16.32	0.1795	Not significant
Pure error	0.0033	1	0.0033			
Cor total	1.45	9				

The relationship between the variance of regression and the residual variance yields Fisher's *F* value (*F* value = *S*2 reg/*S*2 err). With an *F*-value of 6.28, the model appears to be significant, and there is only a 4.96% chance that noise could have caused such a high *F*-value. Since their *p*-values were less than 0.0500, significant model terms were determined to be *A* and *A*^2^. Removing model terms that have *p*-values higher than 0.1000, which are deemed insignificant, while preserving the model hierarchy, could enhance the model's functionality. Furthermore, the Lack of Fit *F*-value of 16.32 indicates that there is a 17.95% possibility that the value is due to noise, indicating that the lack of fit is not significant relative to the pure error. It is desirable that the model fits the data well, as evidenced by this non-significant lack of fit.

#### 3.4.2 Actual and predicted gas sensor sensitivity to methane

**Fig. 9:**
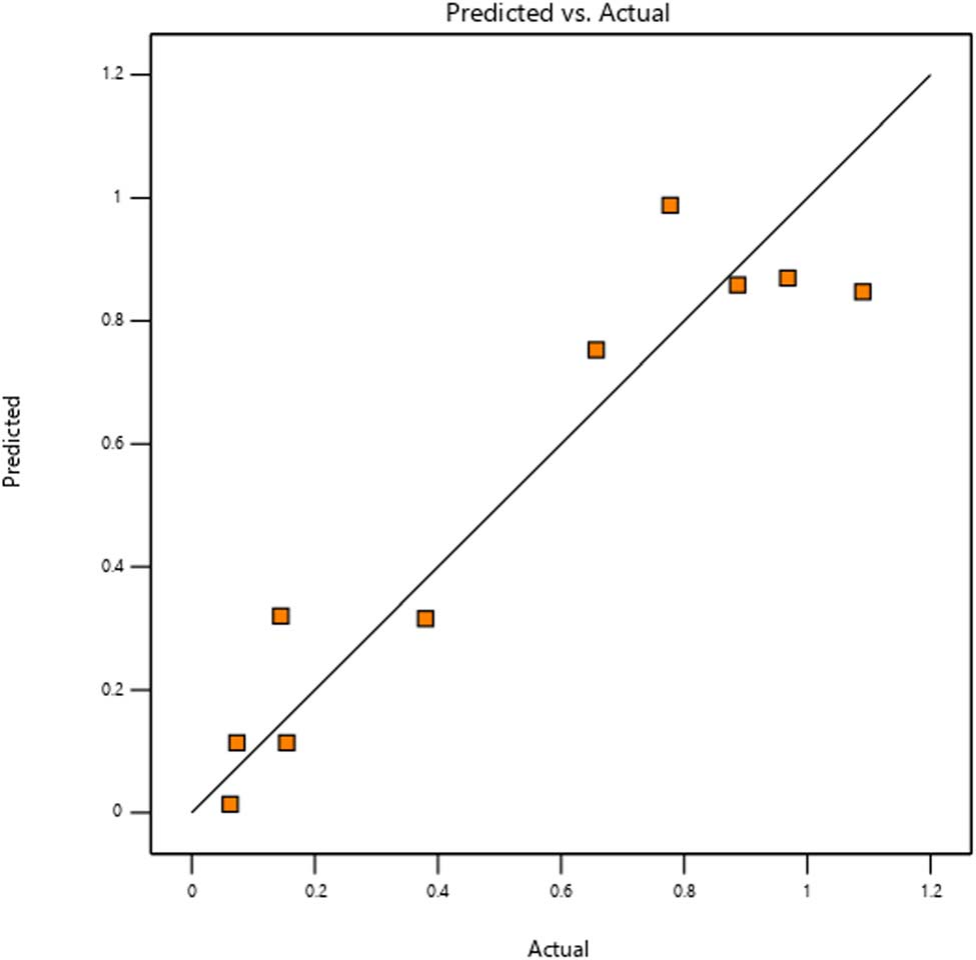
Predicted *vs.* actual value plot for ZnO-G gas sensor's sensitivity.

The actual and the predicted sensitivity values of ZnO-G gas sensors to methane are plotted in Fig. 9. The predicted values were obtained from the model and produced using the approximation functions, while the actual values are the measured response data for a specific run. Strong agreement between the actual and predicted values would be shown by data points that closely coincide with the diagonal line in an ideal model. Although several data points in this study are very close to the line, there are also some significant deviations, which emphasize the limited prediction ability of the model. In comparison to the *R*^2^ and adjusted *R*^2^ values, the predicted *R*^2^ value of 0.2038 suggests a lower ability to predict responses. These variations imply that although the model identifies important patterns, it might not fully explain all the variability in the data. Refinement of the model, such as fixing possible overfitting or adding more experimental data, may increase the predictive accuracy and general dependability of the model. However, the predicted ability in certain cases aligns closely with the line at several points, indicating that the model successfully captures key trends in the data and provides a solid basis for further optimization and refinement to improve its predictive performance.

#### 3.4.3 Gas sensor sensitivity to methane

**Fig. 10:**
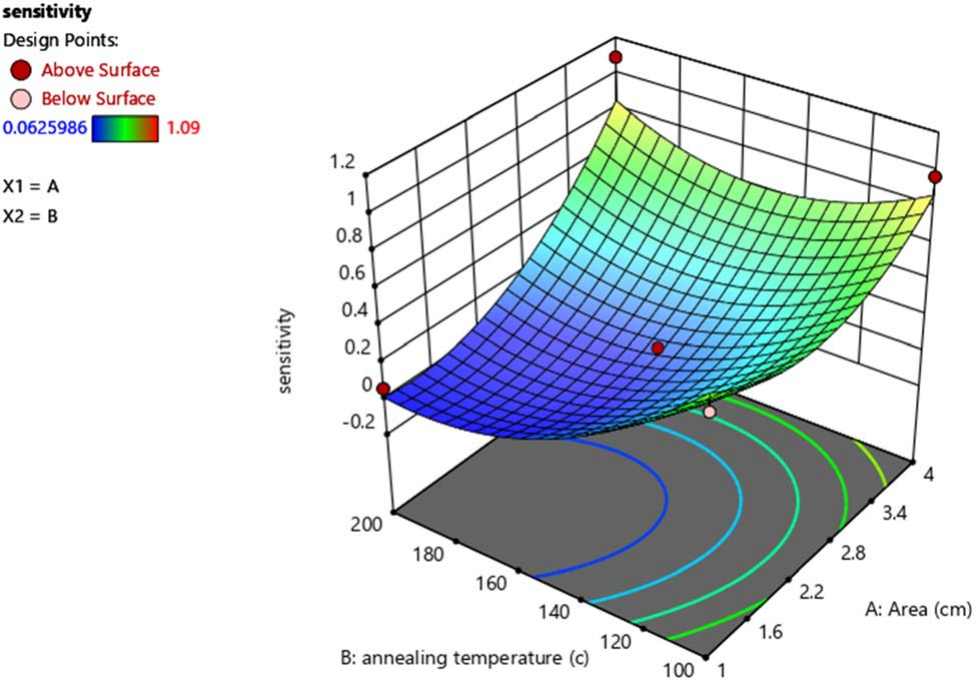
Three-dimensional plots of sensitivity based on the interactive effects of area and annealing temperature.

In order to investigate how the two parameters affected the detection of methane, three-dimensional plots were generated using response surface methodology. According to the ANOVA results, the area of the sensing layer had the biggest effect on methane sensitivity, while annealing temperature had the least impact on the sensor response. This is supported by the highest response observed at the data point corresponding to 4 cm^2^ in the response surface graph, and by the low *p*-value of 0.0023 for the area compared to annealing temperature, which has a *p*-value higher than 0.05. Fig. 10 displays the three-dimensional response surfaces that were built to illustrate the two most crucial factors (area of the sensing layer and annealing temperature) on methane detection.

#### 3.4.4 Process optimization

In the detection of methane using a ZnO-G gas sensor, one of the main aims of this study was to find the optimum process parameters that provide relatively high sensitivity yields and economic feasibility, and the most important property of a gas sensor is its response speed. For the gas sensor to be economically viable, it must have a high sensitivity capacity. The two factors listed in Table 7 are used to optimize this response. The function of desirability was applied using Design-Expert software. The most desirable experimental settings were chosen for verification. Together with the predicted and experimental sensitivity values of the ZnO-G gas sensor, methane gas was detected under the experimental conditions listed in Table 8.

**Table 7:** Constraints for the optimization of sensitivity for the ZnO-G gas sensor

Name	Goal	Lower limit	Upper limit	Lower weight	Upper weight	Importance
*A*: area (cm^2^)	In the range	1	4	1	1	3
*B*: annealing temperature (°C)	In the range	100	200	1	1	3
Sensitivity	Maximize	0.0625986	1.09	1	1	3

**Table 8:** Model validation

*A*: area (cm^2^)	*B*: annealing temperature (°C)	Sensitivity	
		Predicted	Experimental
4	100	0.870	0.968

The predicted and experimental sensitivity values for the specific preparation condition (area of 4 cm^2^ and an annealing temperature of 100 °C) are displayed in the model validation table (Table 8). The measured sensitivity was 0.968, whereas the predicted sensitivity value was 0.870. The small deviation between the predicted and experimental values (10%) demonstrates that the model created using Response Surface Methodology (RSM) is dependable and capable of accurately predicting the sensitivity response.

**Fig. 11:**
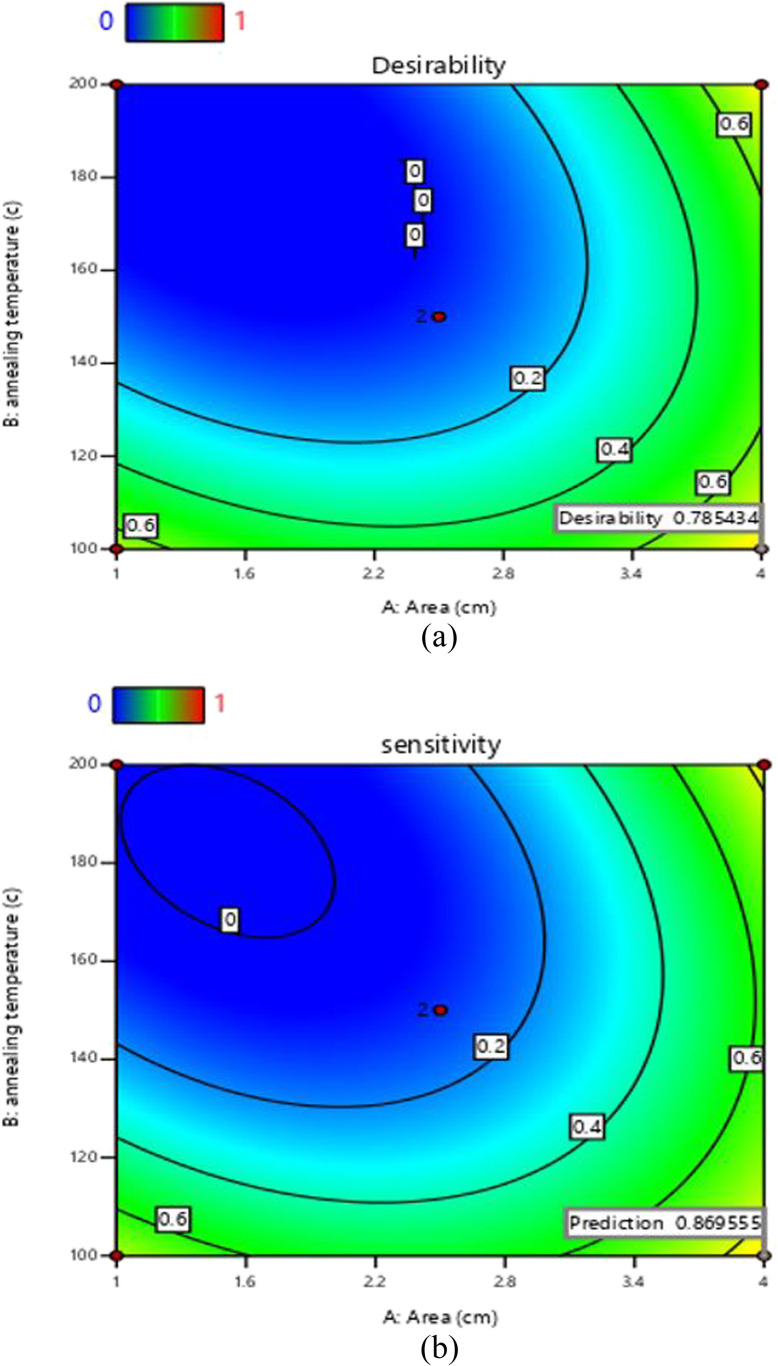
Optimum region of the area and annealing temperature for sensor sensitivity: (a) desirability factor and (b) target response.

The illustration in Fig. 11 shows the outcomes of optimizing the gas sensor response depending on two parameters: area of the sensing layer (cm^2^) and annealing temperature (°C). In Fig. 11(a), the desirability plot shows how close the proposed conditions are to reaching the target response. The likelihood of getting the best response is indicated by the desirability factor, which is scaled from 0 to 1. The utility levels are shown by the colored contours, where areas closer to 1 are more desirable. In this study, the desirability is 0.785, which demonstrates the model's capability to produce the best response. The sensitivity plot in Fig. 11(b) shows the ideal circumstances for the gas sensor's response. For the factors (area of the sensing layer and annealing temperature), the contour lines show the range of response values. The lines' curvature indicates that these parameters interact significantly. Stronger interactions are shown in regions with denser or more curved arcs, highlighting the importance of combining both elements for the best sensor performance. In this study, a sensitivity of 0.870 was found, as the curvy arcs demonstrate higher sensitivity.

### 3.5 Comparison of the proposed study with other researches

Table 9 shows the data comparison of the gas sensor with those reported in the literature for methane detection. The data have been compared based on methane exposure at the same concentration, which is 5000 ppm. A study reported in ref. 2 highlighted a ZnO gas sensor, with the dipping method as the deposition technique, fabricated on an alumina substrate, producing a response value of 20.577% at 5000 ppm of methane concentration. Next, the SnO_2_ gas sensor fabricated using the screen printing method on alumina substrates, as emphasized in ref. 42 produced a response value of 10% at operating temperatures ranging from 300 °C to 400 °C. As indicated in ref. 43 the ZnO/Pd@ZIF-8/Pt sensor produces the greatest response value of 304.6% at 5000 ppm of methane exposure. This sensor was created on a ceramic substrate using the coating method at an operating temperature of 230 °C. Meanwhile, in ref. 44 the SnO_2_–Pt gas sensor has a response value of 130% at 5000 ppm of methane concentration, wherein the gas sensor was fabricated using the sputtered technique on the thermally oxidized Si wafer at an operating temperature of 400 °C. In this study, the ZnO-G gas sensor was created using the screen printing technique on Kapton film substrates at room temperature, generating a response value of 3.1585% at 5000 ppm of methane concentration. In comparison, the ZnO/Pd@ZIF-8/Pt gas sensor had the greatest response value (304.6%) among the sensors due to its advanced hybrid structure. On the other hand, the SnO_2_–Pt gas sensor, which was fabricated by the sputtering method on a thermally oxidized silicon wafer, shows a 130% response at 400 °C. Although its design demonstrates a higher response value, it requires higher operating temperatures (400 °C). Similarly, the SnO_2_ gas sensor, which was fabricated by screen printing on an alumina substrate at high operating temperatures between 300 and 400 °C, however, has a relatively low response value which is 10%. In contrast, the ZnO gas sensor made by dipping on alumina substrates produced a moderate sensitivity of 20.577% at room temperature. In the context of this study, the ZnO-G gas sensor in development phase produced a relatively low in response value (3.1585%); however, this sensor is especially well-suited for flexible, low-power applications, fulfilling the needs of systems that are portable and energy efficient.

**Table 9:** Comparison data from past research

Sensing material	Deposition technique	Substrate	Operating temperature	Response value at 5000 ppm of CH_4_ concentration	Ref.
ZnO	Dipping method	Alumina	Room temperature	20.577%	2
SnO_2_	Screen printing	Alumina	300–400 °C	10%	42
ZnO/Pd@ZIF-8/Pt	Coating method	Ceramic	230 °C	304.6%	43
SnO2–Pt	Sputtering technique	Thermal oxide Si wafer	400 °C	130%	44
ZnO-G	Screen printing	Kapton film	Room temperature	3.1585%	This study

## 4 Conclusion

In conclusion, the ZnO-graphene gas sensor was successfully fabricated using the screen-printing technique on Kapton films at room temperature. The gas sensor exhibited good response to methane. Furthermore, the fabricated ZnO-G gas sensor also showed good response and sensitivity at room temperature, where the sensor demonstrated an increase in response as the methane concentration increased, thus increasing the sensitivity of the sensor. Finally, the optimization of the ZnO-G was successfully conducted by investigating two parameters, which are the area of the sensing layer and annealing temperature. The optimization demonstrates the experiment model in quadratic, with an *R*^2^ value of 0.8871, demonstrating the values close to 1, which indicates the experimental data and the quadratic model have a significant degree of fit. The result showed that the area of the sensing layer has a less significant influence on the model, with a low *p*-value of 0.0023 compared to annealing temperature, which has a *p*-value higher than 0.05. According to the higher desirability (0.785) and sensitivity plot (0.870), an area of the sensing layers of 4 cm^2^ and annealing temperature of 100 °C indicate the optimal parameters to achieve higher sensitivity of the ZnO-G gas sensor. Overall, the proposed model in this study is significant, and the optimization of methane sensing response was successfully implemented using the RSM model. Efficient detection and monitoring of methane are essential for mitigating safety risks and preventing environmental pollution. The fabricated ZnO-G can be reproduced for methane sensing applications at room temperature, such as for the detection of methane in biomass, methane leakage detection in renewable energy, or the detection of methane emission from industrial processes. For future work, the number of samples used in experimental data will be increased to obtain the predicted *R*^2^ (0.2038) value and an adjusted *R*^2^ (0.7459) value closer to 1 to enhance methane sensing performance.

## Conflicts of interest

There are no conflicts to declare.

## Data Availability

The data that support the findings of this study are available from the corresponding author, Siti Amaniah Mod Chachuli, upon reasonable request.
